# Supplementary material for: “This is not my body”: Therapeutic experiences and post-treatment health of people with rifampicin-resistant tuberculosis
Source: PLoS One. 2021 Oct 18;16(10):e0251482. doi: 10.1371/journal.pone.0251482 (PMC8523213; doi:10.1371/journal.pone.0251482)
Supplement: S1 File — (DOCX) [file pone.0251482.s002.docx]

**APPENDIX 1: IN DEPTH INTERVIEW TOPIC GUIDE**

**Investigating survival and TB recurrence after successful drug-susceptible- and multidrug/rifampicin -resistant TB treatment**

**_____________________________________________________________________________________**

**Unique study no**: _______________________ **Gender: (M or F):** __________

**DS- or DR-TB:**  _____________________ **Study location: (KZN or WC):** _____________

**Interviewer:** ___________________________ **Date:** __­­­___________

**Questions:**

1. How many years ago did you start MDR-TB treatment?
2. Can you remember and describe how sick you were when you started MDR-TB treatment?

**Probes**

- Were you able to walk when you were first diagnosed with TB?
- Were you admitted to hospital at the time?
- When you started MDR-TB treatment how long were you admitted to hospital for?
- How long have you been in hospital for this time?
- Were you admitted to hospital at any other time during your TB treatment?
- Have you been able to keep doing your normal household responsibilities?
- Were you working when you got MDR-TB? Did you have to stop working?

1. Can you describe how MDR-TB and its treatment affected your life whilst you were taking the treatment?

**Probes**

- How did you feel after you took all the medication?
- Did the medicine affect you in any way?
- How long after the treatment started did you start to feel better?
- Were you tempted to stop taking the treatment at any time?
- Did taking the treatment affect your family life, community life or work life in any way?

1. Can you describe how the MDR-TB and its treatment have affected your life?

**Probes**

- Physical: Are you still able to do the same things physically? Such as walk the same distance? Run to catch a bus? Pick up a child?
- Mental: Are you more anxious or fearful than you were before you had TB? Do things worry you more?
- Relationships: Were any of your relationships with your close family or friends affected by having TB? What happened?
- Stigma/discrimination? Has anyone or any group in your wider circle of acquaintances avoided you since you had TB?

1. Is there anything you can no longer do since you had MDR-TB and started TB treatment? If yes, please can you give some examples?
2. Do you ever worry that you might get MDR/RR-TB again? Can you explain more about this?

**Open attitude question:**

We know that MDR/RR-TB treatment is lengthy and has many side-effects. Since completing treatment, can you explain to me how you feel in relation to your overall health? You have completed MDR/RR-TB treatment, do you feel how you did before you were diagnosed with MDR/RR-TB or are there any differences in your health or quality of life after treatment was completed? Please explain. Have you had a recurrence of MDR/RR-TB?

**Probes**

- Does participant feel they have enough strength to carry out day-to-day activities?
- Does participant feel their health status is static or does it continue to improve since they completed treatment?
- Anxieties about the return of MDR/RR-TB
- Any illnesses due to MDR/RR-TB treatment
- General health status after treatment completion
